# Ion transport activity and optogenetics capability of light-driven Na^+^-pump KR2

**DOI:** 10.1371/journal.pone.0256728

**Published:** 2021-09-10

**Authors:** Shoko Hososhima, Hideki Kandori, Satoshi P. Tsunoda

**Affiliations:** 1 Department of Life Science and Applied Chemistry, Nagoya Institute of Technology, Showa-ku, Nagoya, Japan; 2 OptoBio Technology Research Center, Nagoya Institute of Technology, Showa-ku, Nagoya, Japan; 3 PRESTO, Japan Science and Technology Agency, Kawaguchi, Saitama, Japan; Indiana University School of Medicine, UNITED STATES

## Abstract

KR2 from marine bacteria *Krokinobacter eikastus* is a light-driven Na^+^ pumping rhodopsin family (NaRs) member that actively transports Na^+^ and/or H^+^ depending on the ionic state. We here report electrophysiological studies on KR2 to address ion-transport properties under various electrochemical potentials of Δ[Na^+^], ΔpH, membrane voltage and light quality, because the contributions of these on the pumping activity were less understood so far. After transient expression of KR2 in mammalian cultured cells (ND7/23 cells), photocurrents were measured by whole-cell patch clamp under various intracellular Na^+^ and pH conditions. When KR2 was continuously illuminated with LED light, two distinct time constants were obtained depending on the Na^+^ concentration. KR2 exhibited slow ion transport (τ_off_ of 28 ms) below 1.1 mM NaCl and rapid transport (τ_off_ of 11 ms) above 11 mM NaCl. This indicates distinct transporting kinetics of H^+^ and Na^+^. Photocurrent amplitude (current density) depends on the intracellular Na^+^ concentration, as is expected for a Na^+^ pump. The M-intermediate in the photocycle of KR2 could be transferred into the dark state without net ion transport by blue light illumination on top of green light. The M intermediate was stabilized by higher membrane voltage. Furthermore, we assessed the optogenetic silencing effect of rat cortical neurons after expressing KR2. Light power dependency revealed that action potential was profoundly inhibited by 1.5 mW/mm^2^ green light illumination, confirming the ability to apply KR2 as an optogenetics silencer.

## Introduction

Microbial rhodopsins are retinal-binding membrane proteins which function as ion-transporters, photo-sensor and light-regulated enzymes [1]. NaR is one of the microbial rhodopsins that actively transports Na^+^ and H^+^ depending on ionic conditions [2, 3]. Since the discovery of KR2, an NaR first identified from a marine flavobacterium *Krokinobacter eikastus*, its photochemical properties have been intensively investigated by spectroscopy. Upon photoisomerization of all-*trans* retinal, KR2 undergoes a photocycle which involves K, L, M and O intermediates with different absorption maxima and different protein conformation. It has been proposed that Na^+^ is taken up from the cytoplasmic side after the M intermediate forms and released as the O intermediate to the extracellular side [2, 4].

Two structural studies revealed the detailed molecular architecture of KR2 and provided insight into its ion transporting mechanism [5, 6]. One of the important issues was how Na^+^ can efficiently move over the chromophore region where a large electric barrier exists. This was explained by the observation that after photoisomerization, the protonated Schiff-base is neutralized because the positive charge is caught by a neighboring D116, allowing Na^+^ to pass through. Another important aspect in those structural studies was that Na^+^ is unbound in rhodopsin upon an energy input (light absorption), whereas usual active pumps are energized in the substrate-bound state. Thus, NaR has a very unique active transport mechanism. We recently described this as an alternative Panama canal model [3]. MD simulations and QM/MM calculations modeled how conformation dynamics contribute to Na^+^ pumping [7]. That study proposed three putative Na^+^ binding sites.

Notably, NaRs transport not only Na^+^, but also H^+^ depending on ionic conditions. Flash photolysis measurement of KR2 under various pH and Na^+^ conditions revealed that the M-decay rate in the absence of Na^+^ is 8000 times faster than that in the presence of Na^+^ [8]. The ion transporting ratio between H^+^ and Na^+^ varies among NaR subfamily members, as demonstrated by an experiment using a pH electrode after expressing these NaRs in *E*. *coli* [9]. KR2 transports both Na^+^ and H^+^ in significant amounts whereas H^+^ pumping activity is very weak or almost zero in FdNaR and NyNaR, while retaining reasonable Na^+^ pumping signals. This indicates that the latter two NaRs might be more selective to Na^+^ than KR2.

We previously conducted electrophysiological studies of several NaRs, including KR2, FdNaR, and others [9]. Although the photocurrent amplitudes largely depended on the NaR genes, and/or tagged-fluorescent proteins, we were able to measure the pumping current from several NaRs. Basic properties of NaRs such as current-voltage relation and action spectrum (wavelength dependency) have been studied for FdNaR, NyNaR and SrNaR [9].

Other research groups have engineered chimeric NaRs and modified KR2 to enhance their pumping functions and characterized those pumping properties by electrophysiology and spectroscopy [10–12]. In particular, Grimm *et al*. investigated effect of ionic conditions (dpH, d[Na^+^]) and membrane voltage (dΦ) on the KR2 pumping activity [11]. Proton pumping rhodopsins such as BR and ones from *Acetabularia*, *Gloeobacter* and *Chlorella* have been studied by electrophysiology more extensively [13–16]. Among these, it has been shown to possess an additional blue-shifted photocycle which exhibits non-net proton flow. It remains unknown whether such non-transporting cycle exists in NaRs too.

In this report, we used KR2 fused with eYFP and signal peptides (membrane trafficking signal and ER export signal) to improve the membrane expression level, and further characterized the photocurrent properties. In addition, NaR is an efficient optogenetics silencer in cultured neurons and animals [5, 9, 11]. It would be informative to know the minimum light intensity required for neuronal silencing by KR2. Thus, we investigate the light-power dependency of KR2 in cultured neurons.

## Materials and methods

### Expression plasmids

phKR2-3.0-eYFP and pCamKIIa-hKR2-3.0-eYFP were kind gifts from Dr. H. Yawo (Tohoku University, Japan). Details of the constructs have been described in [4] and [9].

### Cell culture

The electrophysiological assays of KR2 were performed on ND7/23 cells, hybrid cell lines derived from neonatal rat dorsal root ganglia neurons fused with mouse neuroblastoma [17]. ND7/23 cells were grown on a collagen-coated coverslip in Dulbecco’s modified Eagle’s medium (Wako, Osaka, Japan) supplemented with 2.5 μM all-*trans* retinal, 10% fetal bovine serum (Biowest, Nuaille, France) under a 5% CO_2_ atmosphere at 37°C. The expression plasmids were transiently transfected by using Lipofectamine 2000 (Invitrogen, Carlsbad, CA, USA) according to the manufacturer’s instructions. Electrophysiological recordings were then conducted 24–36 h after transfection. Successfully transfected cells were identified by eYFP fluorescence under a microscope prior to the measurements.

Cortical neurons were isolated from embryonic day 16 Wistar rats (Charles River Laboratories Japan, Inc., Yokohama, Japan) using Nerve-Cells Dispersion Solutions (Wako, Tokyo, Japan) according to the manufacturer’s instructions and grown in culture medium (Wako) under a 5% CO_2_ atmosphere at 37°C. The expression plasmids were transiently transfected in cortical neurons by calcium phosphate transfection at 5 or 7 days *in vitro* (DIV). Electrophysiological recordings were then conducted at DIV21 on neurons identified as expressing eYFP fluorescence under a conventional epifluorescence system [18].

### Electrophysiology

All experiments were carried out at room temperature (22±2°C). Photocurrents were recorded as previously described using an Axopatch 200B amplifier (Molecular Devices, Sunnyvale, CA, USA) under a whole-cell patch clamp configuration [9]. The data were filtered at 5 kHz (for recording with LED), or 10 kHz (for recordings with a flash laser), and sampled at 20 kHz (Digdata1550, Molecular Devices, Sunnyvale, CA, USA) and stored in a computer (pClamp10.6, Molecular Devices). All patch-clamp solutions are described in Table 1. Osmolality of all solutions was adjusted to 300 mOsm by adding an appropriate amount of glucose. The liquid junction potential was calculated and compensated by pClamp 10.6 software. Time constants were determined by a single exponential fit unless noted otherwise. In the case of a double exponential fit, the apparent time constant was calculated as described previously [19].

**Table 1:** Composition of bath and pipette solutions in experiments with ND7/23 cells and neurons.

Recording for ND7/23 cells		**pH**	**NaCl**	**NMG**	**MgCl**_**2**_	**CaCl**_**2**_	**HEPES**	**Tris-HCl**	**EGTA**	**Glucose**	
**Bath**	Stadard	7.4	140	0	2	2	10	0	0	11	
**Pipette**	Standard	7.4	110	0	2	1	10	0	10	3	
	110 mM NaCl, pH 8.0	8.0	110	0	2	1	10	0	10	3	
	110 mM NaCl, pH 9.0	9.0	110	0	2	1	0	10	10	3	
	0 mM NaCl, pH 8.0	8.0	0	110	2	1	10	0	10	3	
	0 mM NaCl, pH 9.0	9.0	0	110	2	1	0	10	10	3	
	11 mM NaCl	7.4	11	99	2	1	10	0	10	3	
	1.1 mM NaCl	7.4	1.1	108.9	2	1	10	0	10	3	
	0.11 mM NaCl	7.4	0.11	109.89	2	1	10	0	10	3	
	0 mM NaCl	7.4	0	110	2	1	10	0	10	3	
Recording for Neuron		**pH**	**NaCl**	**K-gluconate**	**KCl**	**MgCl**_**2**_	**CaCl**_**2**_	**HEPES**	**EGTA**	**Glucose**	
**Bath**	Tyrode’s	7.4	138	0	3	1	2	10	0	11	4 NaOH
											0.02 DNQX
											0.025 D-AP5
											0.1 Picrotoxin
**Pipette**	K-gluconate	7.4	10	125	0	1	0	10	0.2	0	3 MgATP
											0.3 Na_2_GTP
											10 Na_2_-phosphocreatine
											0.1 Leupeptin

Abbreviations: NMG, N-Methyl-D-glucamine; HEPES, 4-(2-hydroxyethyl)-1-piperazineethanesulfonic acid; Tris, tris(hydroxymethyl)aminomethane; EGTA, ethylene glycol tetraacetic acid; DNQX, 6,7-Dinitroquinoxaline-2,3-dione; D-AP5, D-(-)-2-amino-5-phosphonopentanoic acid. All concentrations are in mM.

Action potentials were recorded using an amplifier IPA (Sutter Instrument, Novato, CA, USA) under a whole-cell patch clamp configuration. The data were filtered at 5 kHz and sampled at 10 kHz and stored in a computer. Pipette resistance was 5–10 MΩ. All patch-clamp solutions are described in Table 1. The directly measured liquid junction potential was 16.3 mV and was compensated.

### Optics

**Fig 1:**
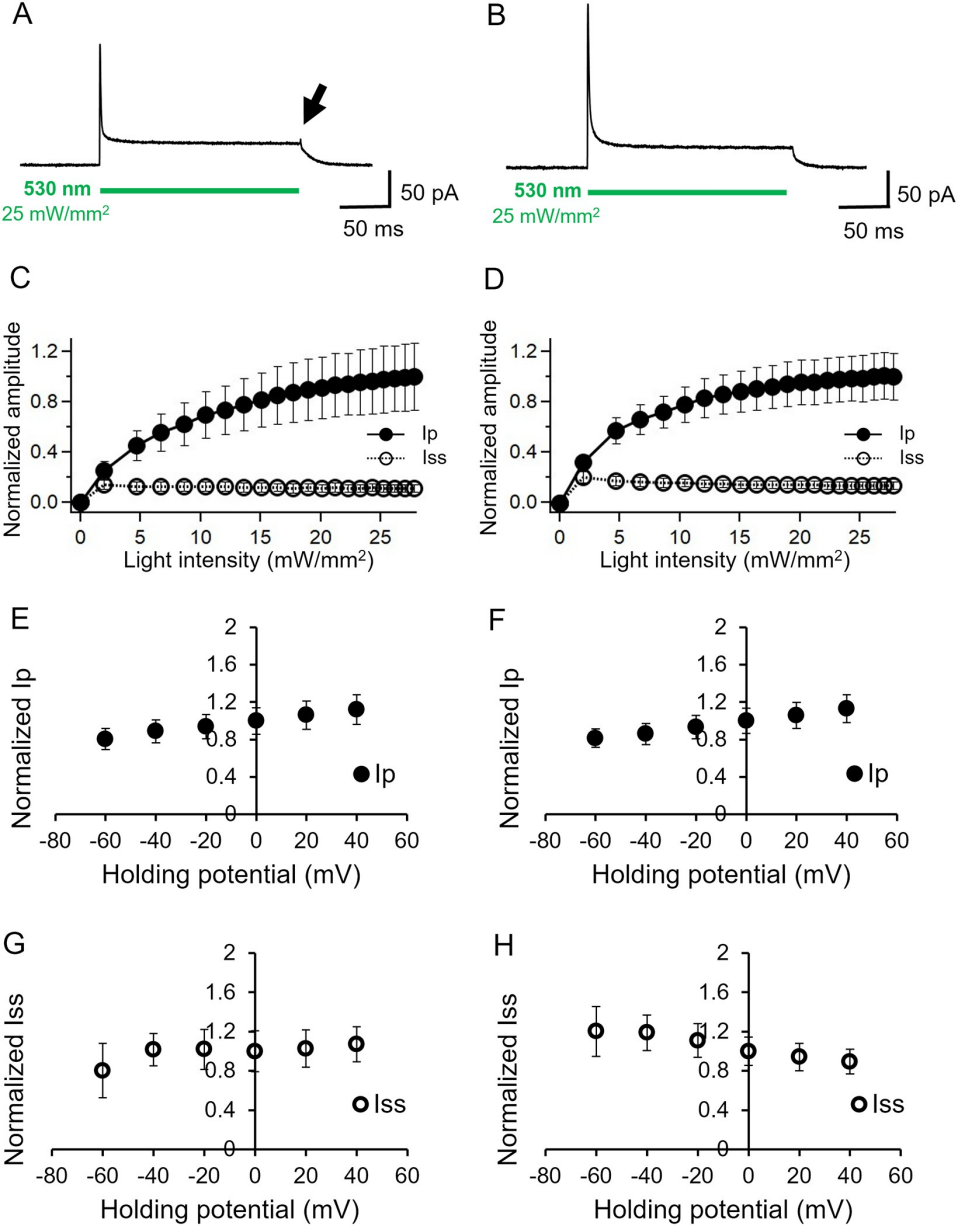
Basic characterization of KR2 photocurrent in ND7/23 cells. Representative photocurrent at 0 mV in the absence (A) and presence (B) of 110 mM NaCl in intracellular solution while the bath solution contained 140 mM NaCl. pHs were adjusted at 7.4 for both solutions. See Table for more details. 530 nm light (25 mW/mm^2^) was illuminated for 200 ms as the green bar indicates. C and D, light power dependency on the peaks (I_p_) and steady state (I_ss_) photocurrent, in the absence and presence of NaCl intracellular solution, respectively (n = 7). E and F, Current-voltage relation (I/V plot) for the peak current (n = 12, 9). The currents were normalized to the value at 0 mV. G and H, Current-voltage relation (I/V plot) for the steady state component. The currents were normalized to the value at 0 mV (n = 12, 9).

**Fig 2:**
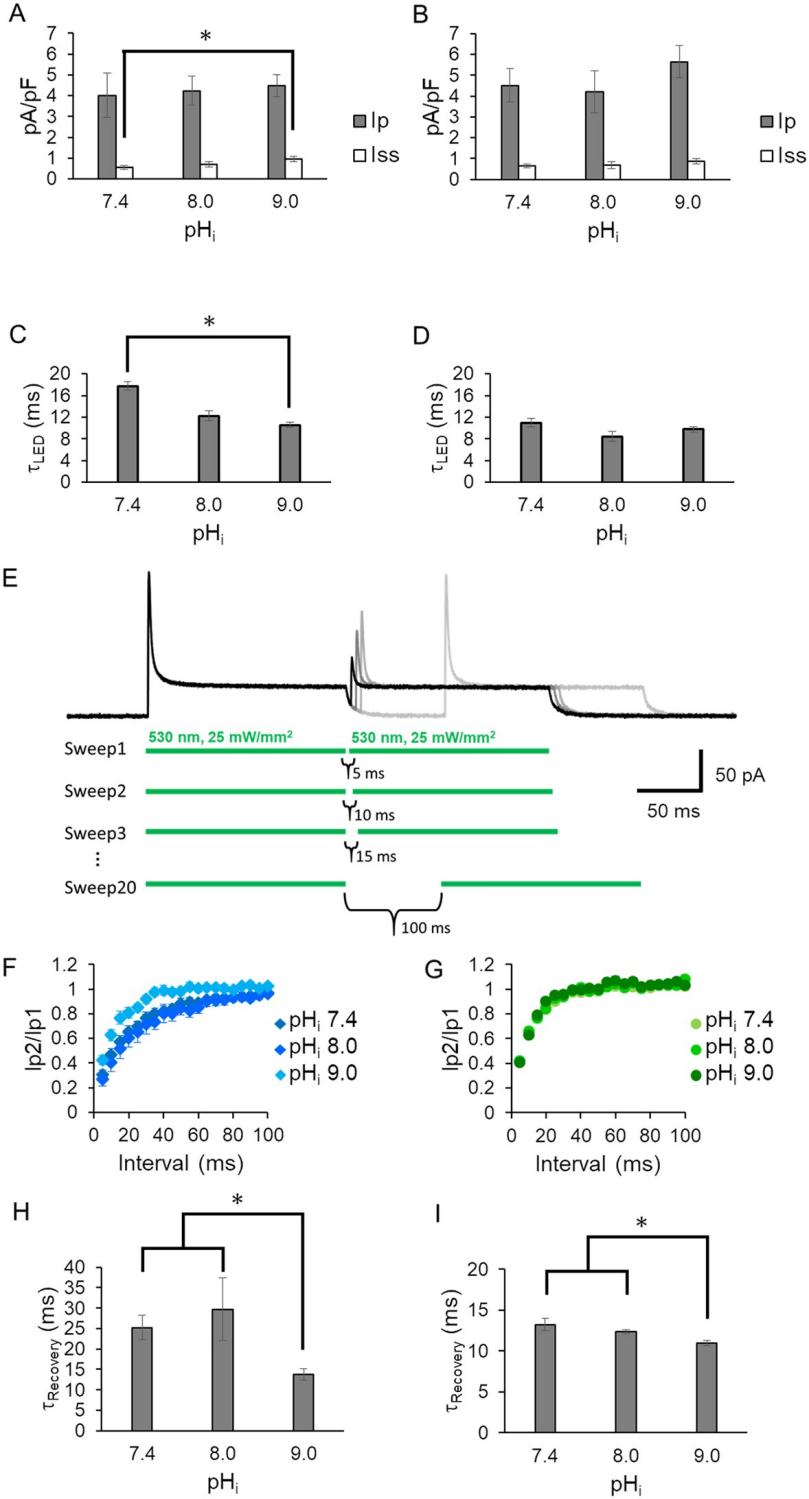
Effect of intracellular pH (pH_i_). Photocurrent amplitude at 0 mV under three pH_i_ conditions (7.4, 8.0 and 9.0) in the absence (A) and presence (B) of 110 mM NaCl inside (n = 6–14, *p<0.05). The bath solution contained 140 mM NaCl at pH 7.4. See Table for more details. Gray bar: peak current (I_p_); white bar: steady state current (I_ss_). Off kinetics value (τ_off LED_) under 3 pH_i_ conditions in the absence (C) and presence (D) of 110 mM NaCl in intracellular solutions. E, Representative KR2 photocurrent in double pulse experiment with various dark periods at 0 mV. LED light at 530 nm was applied as indicated by green lines below the photocurrent traces. The dark interval between the two light pulses was prolonged by 5 ms. Photocurrents traces are overlaid in different gray scales. The second peak amplitude was recovered as the dark interval was prolonged while the steady state current remained unchanged. The inter-sweep dark period was always set at 2.0 s to guarantee completed dark adaption. The intracellular solution contained 110 mM NaCl at pH 7.4 while the bath solution contained 140 mM NaCl at pH 7.4. D, Time course of the second peak recovery (n = 6–22) (F and G). The second peak ratio (Ip2/Ip1) was plotted as a function of the dark period (ms). F, The measurement was performed at three different pH_i_ in the absence of NaCl in the intracellular solutions. G, the same experiment as in F but in the presence of 140 mM NaCl inside the cells (n = 6–8). H and I, kinetic values obtained from F and G (* p<0.05) (τ_Recovery_). The recovery time course in F and G was plotted with a single exponential function.

**Fig 3:**
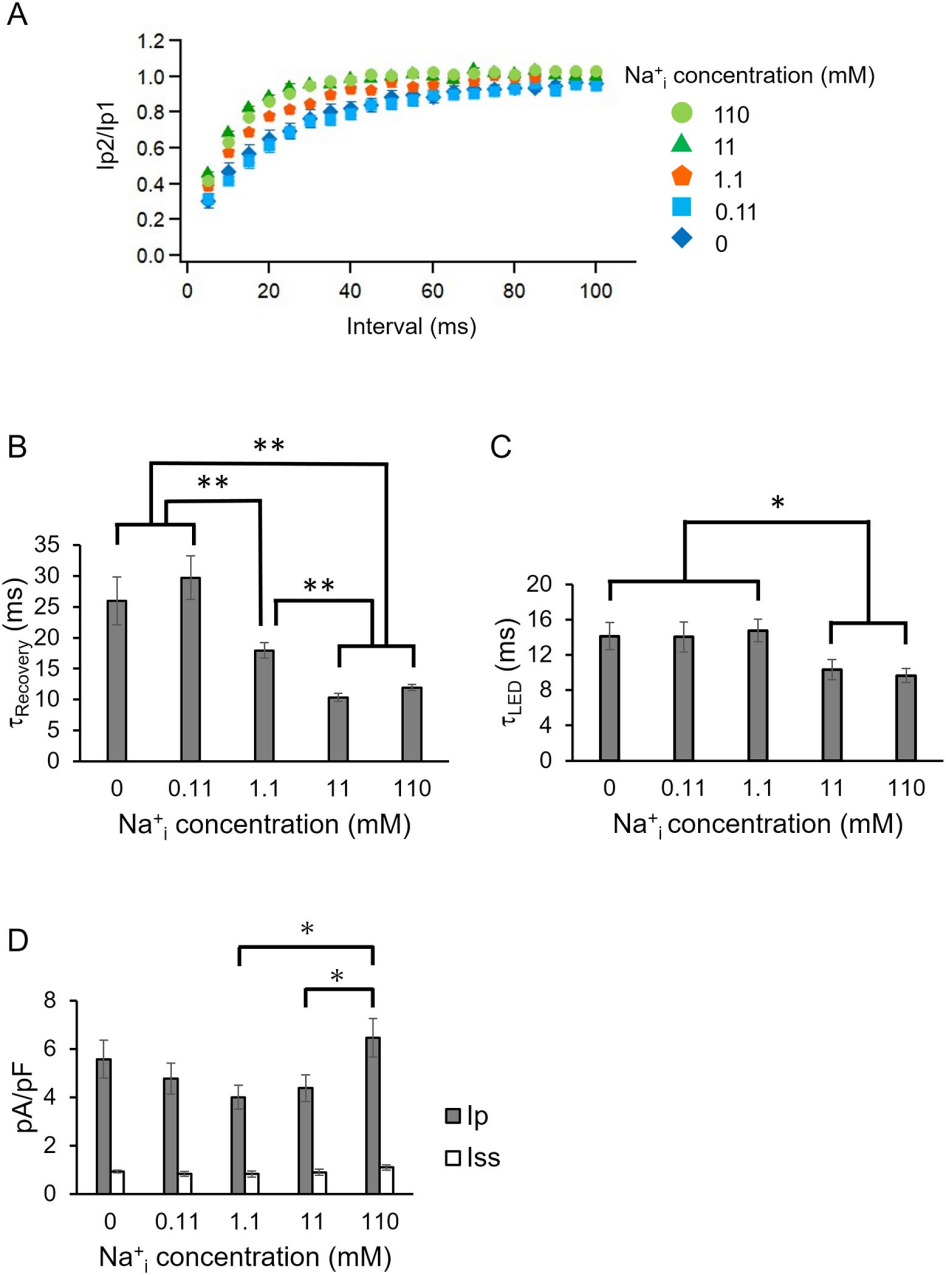
Effect of intracellular Na^+^ concentration. A, The recovery kinetics of the second peak photocurrent in the presence of various NaCl concentrations at pH 7.4 in intracellular solution (n = 6–16). See Table for more details. The same recordings shown in Fig 2E were performed to assess the effect of Na^+^. B, Kinetic parameters (τ_Recovery_) obtained from A (* p<0.05, ** p<0.01). C, Photocurrent kinetics after shutting the LED light (τ_offLED_) was determined under various NaCl concentrations inside the cell. D, Photocurrent amplitude in various NaCl concentrations inside the cell while the bath solution contained 140 mM NaCl (n = 10–14, * p<0.05).

**Fig 4:**
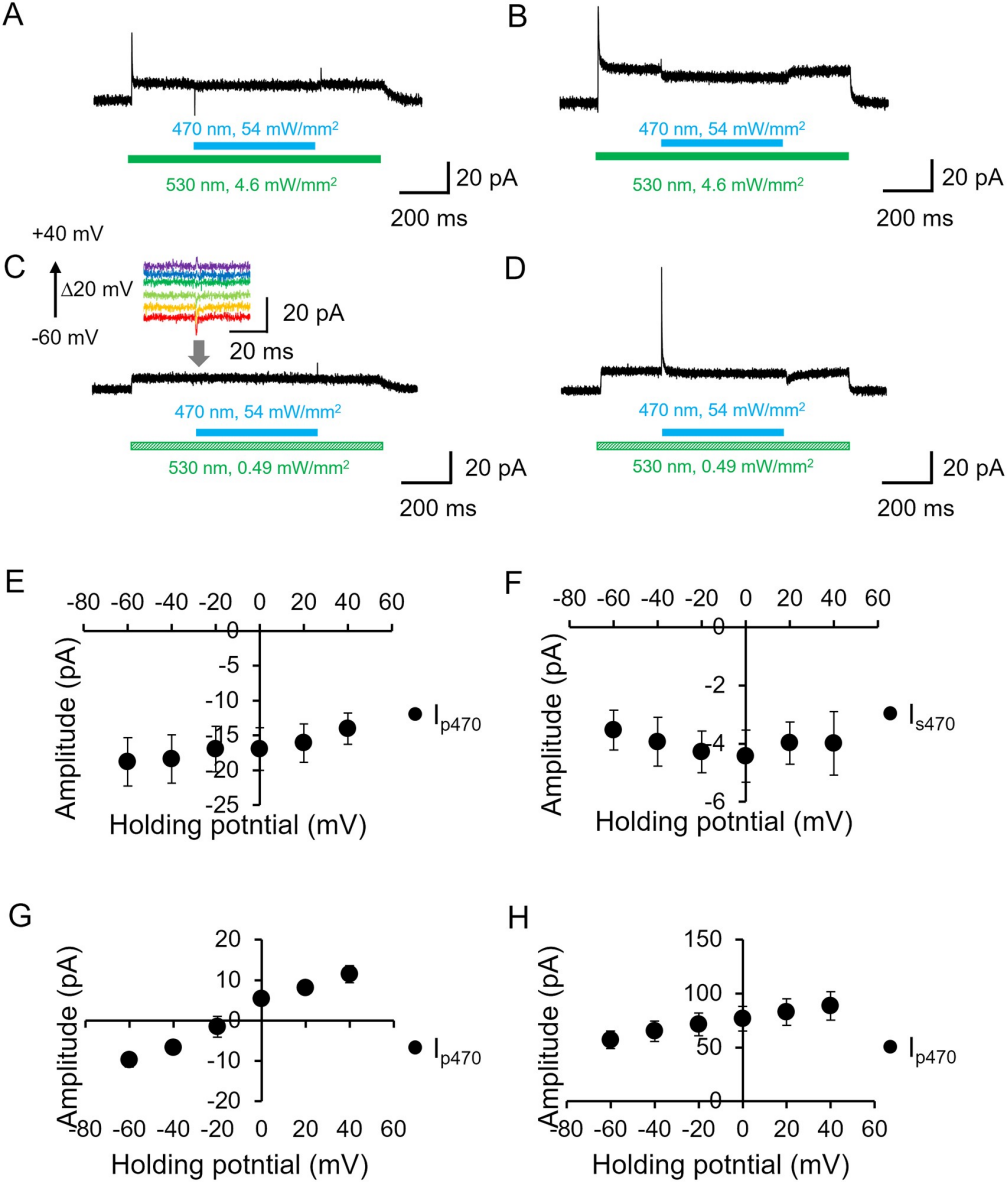
Effect of additional blue light (probing M intermediate). A and B, Representative photocurrent traces in the absence (A) and presence (B) of intracellular NaCl (110 mM) at pH 7.4 while the bath solution contained 140 mM NaCl a pH 7.4. The membrane voltage was clamped at 0 mV. Blue LED (470 nm at 54 mW/mm^2^) was illuminated on top of a green LED illumination (530 nm at 4.6 mW/mm^2^), as shown by colored bars under the current traces. C and D, The recordings as A and B with a green light (530 nm at 0.49 mW/mm^2^) in the absence (C) and presence (D) of intracellular NaCl. The inset in C shows enlarged traces upon blue light illumination at different membrane voltages. E-H, Current-voltage relation (I/V plot) of the photocurrent during additional blue light illumination (n = 6). The peak component Ip_470_ and the steady state level (Is_470_) were plotted. E and G, in the absence of intracellular NaCl. F and H, in the presence of 110 mM NaCl. E and F, Strong green light (6.6 mW/mm^2^). G and H, Weak green light (0.49 mW/mm^2^).

**Fig 5:**
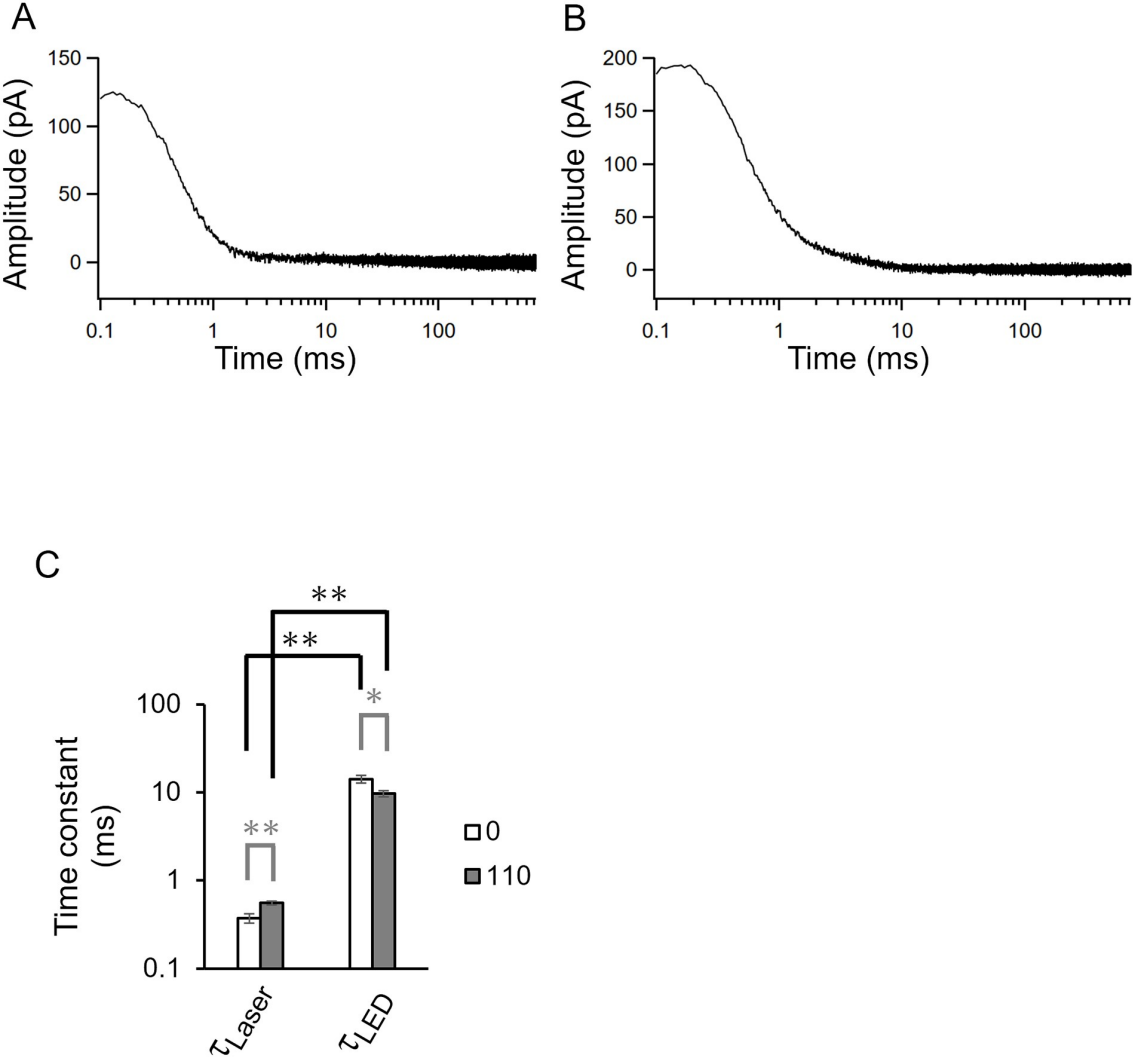
Single photocycle ion transport. Photocurrent of KR2 evoked by a 5 ns flash laser (532 nm) at 0 mV. The recording was performed in the absence (A) and presence (B) of intracellular Na^+^ (110 mM) at pH_i_ 7.4, while bath solution contained 140 mM NaCl at pH 7.4. C, Off-kinetics values obtained from a flash laser (τ_off Laser_), a green LED (τ_off LED_) (n = 7–16, * p<0.05, ** p<0.01). Open bar, in the absence of intracellular Na^+^. Gray bar, in the presence of 110 mM NaCl inside.

**Fig 6:**
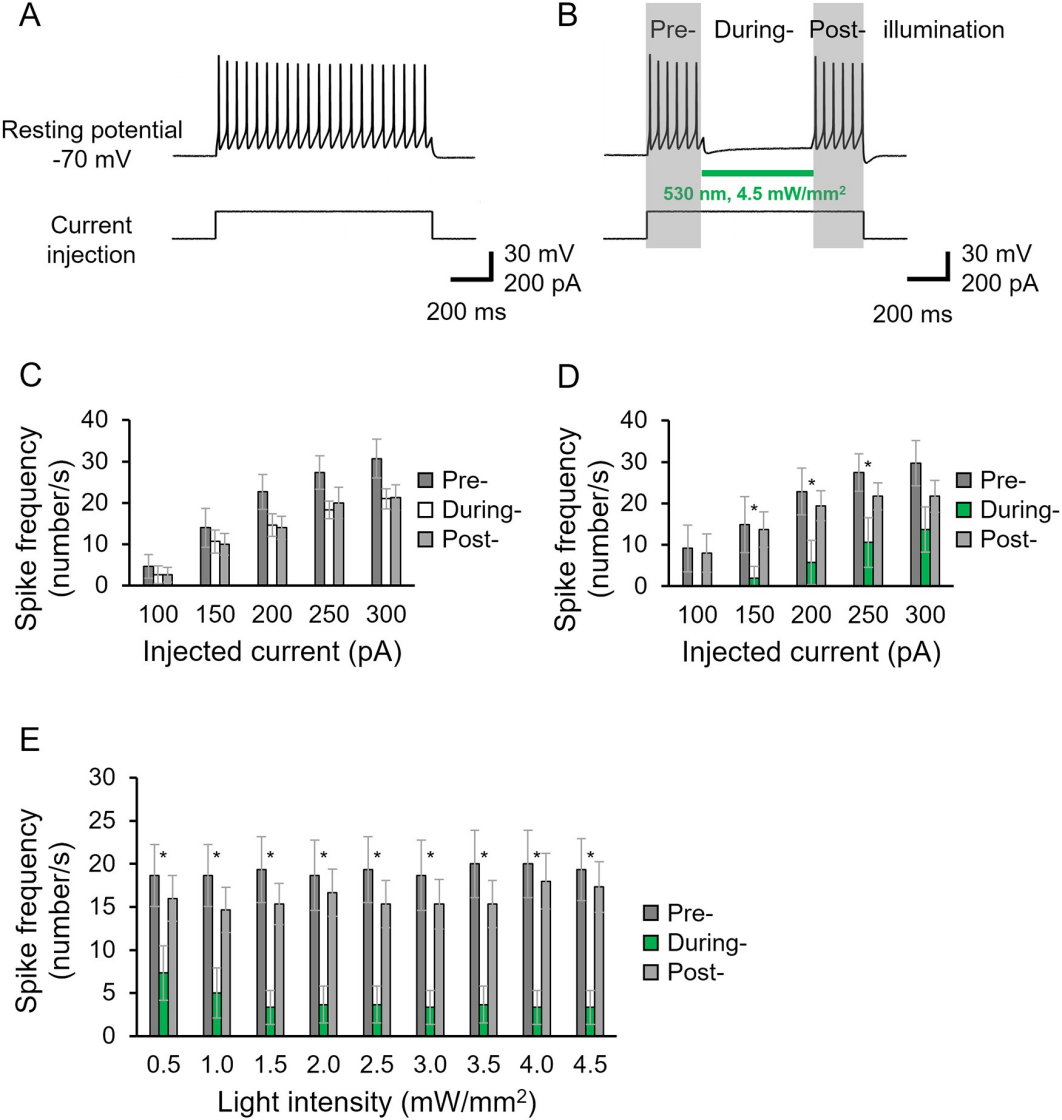
Neuronal silencing by KR2. A, Representative traces of action potentials by current injection (200 pA) as shown below the trace in a KR2-expressing cultured neuron. Solutions used were listed in Table. B, Evoked spikes were suppressed by green light illumination as indicated by a green bar in a KR2-expressing neuron. C and D, Spike frequency at various injected currents are compared in KR2-expressing neurons. Number of spikes in darkness (Pre-illumination), upon illumination (During-illumination) and again in darkness (Post-illumination) are indicated. C, no illumination in “During” shown by white bars. D, illumination in “During” shown by green bars. n = 6, 7. E, light power dependency on neuronal silencing (n = 6). Spike frequency is shown at various light intensities. The neuron was excited by 150 pA current injections.

For the whole-cell voltage clamp (Fig 4), irradiation at 470 or 530 nm was carried out using WheeLED (parts No. WLS-LED-0470-03 or WLS-LED-0530-03, Mightex, Toronto, Canada) controlled by computer software (pCLAMP10.6, Molecular Devices). For the whole-cell voltage-clamp (Figs 1–3 and 5), irradiation at 530 nm was carried out using collimated LED (parts No. LCS-0530-03-22, Mightex) or an ND YAG flash laser, Mini lite 532 nm (Continuum, San Jose, CA, USA). For the whole-cell current-clamp in Fig 6, irradiation at 511 nm was carried out using Colibri7 (Carl Zeiss, Oberkochen, Germany). Light power was directly measured via the microscope objective lens using a visible light-sensing thermopile (MIR-100Q, SSC Inc., Mie, Japan).

### Statistical analysis

All data in the text and figures are expressed as mean ± SEM and were evaluated with the Mann-Whitney *U* test for statistical significance, unless noted otherwise. Means were judged as statistically insignificant when P > 0.05.

### Ethics

This study was approved by the institutional animal care and use Committee (Permission number: 2020001) and carried out according to the Nagoya Institute of Technology Animal Experimentation Regulation.

## Results

We first performed basic characterization of KR2 by a whole-cell patch clamp recording in the absence and presence of Na^+^ in the intracellular solution while the extracellular solution (the bath solution) contained Na^+^ in both conditions. Fig 1A and 1B show representative current traces of KR2 at 0 mV. Illumination at 530 nm and at 25 mW/mm^2^ induced a large peak current (I_p_) which decayed to a low steady state current (I_ss_). The current shape with two components is typically observed in ion-pumping rhodopsins [13–15]. The decay kinetics after shutting-off the light (τ_off LED_) corresponds to the transition from a late intermediate to the dark state. Since the intracellular solution does not contain Na^+^, the photocurrent in Fig 1A must be carried by H^+^, whereas the photocurrent in Fig 1B could be derived from Na^+^ and/or H^+^. The overall current shapes under these two conditions are indistinguishable except for a small positive peak, which was observed when the light was switched off in the absence of Na^+^ (indicated by an arrow in Fig 1A). The peak was more visible when illuminated by light at 470 nm (indicated by an arrow in S1A Fig). Such peak under low Na^+^ conditions was also reported previously [20]. The transient peak could be explained as re-protonation of retinal-Schiff-base during the photocycle of KR2.

Fig 1C and 1D show the light intensity dependence of the photocurrent using an LED (530 nm), near the λ_max_ of KR2. Current amplitude of the peak component (I_p_) increased with a single exponential manner both in the absence and presence of Na^+^ (filled circles). Half-maximal activation was observed at 9.4 mW/mm^2^ in the absence of Na^+^ and 5.5 mW/mm^2^ in the presence of Na^+^. On the other hand, the current amplitude of the steady state current (I_ss_) was already saturated at 2 mW/mm^2,^ (open circles), reproducing the results of a previous study [9]. The half-maximum activation of KR2 has previously been reported as 39.8 mW/mm^2^ for the peak component and 18.7 mW/mm^2^ for the steady state photocurrent [10]. These values are higher than our observations. The differences could be derived from the quality of the light sources, namely we used 530 nm (± 20 nm) light, whereas Hoque *et al*. employed a LED with a broad spectrum range (534–600 nm). Interestingly, the steady state level decreased slightly as the light intensity increased in the presence of Na^+^ which was also observed in a previous report (Fig 1D) [10]. Fig 1E–1H shows the current-voltage relations (I-V plot) of the KR2 photocurrent. The I-V plot of the peak component (I_p_) is linear and almost identical in the presence and absence of Na^+^ (Fig 1E and 1F) in which the current amplitudes increased at a higher membrane potential. This suggests that the initial event of ion transport is accelerated by a membrane depolarization. However, voltage dependency of the steady state levels (I_ss_) showed a non-linear relationship. In the absence of Na^+^, the current amplitude was independent of membrane voltage between -40 and +40 mV while a slight reduction was observed at -60 mV (Fig 1G). Notably the I-V relationship in the presence of Na^+^ was opposed, in which the current amplitude was reduced by about 30% when the membrane voltage rose from -60 mV to +40 mV, indicating that the pumping rate decreased as an electrochemical gradient of Na^+^ to efflux the cell formed (Fig 1H). The I-V plots of all the conditions are summarized in S2 Fig, showing essentially the same tendency as Fig 1E–1H.

Fig 2A and 2B show the pump current at 0 mV with various intracellular pHs (pH_i_) in the absence and presence of intracellular Na^+^ respectively. Both the peak and the steady state currents exhibited no significant dependency on pH_i_ among 7.4, 8.0 and 9.0, except that I_ss_ at pH_i_ 7.4 without Na^+^ was smaller than that of pH_i_ 9.0 (Fig 2A). Considering the substrate concentration (H^+^), the observation cannot be explained. However, τ_off LED_ in pH_i_ 9.0 is smaller than in pH_i_ 7.4, meaning more efficient H^+^ pump at pH_i_ 9.0 (Fig 2C). This could be the reason why I_ss_ in pH_i_ 9.0 is larger than pH_i_ 7.4. τ_off LED_ in the presence of Na^+^ has no significant difference among 3 pH_i_ conditions (Fig 2D).

### Double pulse experiments

We performed a double-pulse recording in which two light pulses were used, separated by dark periods of varying duration to investigate the time-course of recovery of the peak amplitude [14, 15]. Fig 2E shows representative photocurrent traces. As the dark period between the first and second light pulse was prolonged, the peak component (I_p_) of the second light pulse increased. The recovery kinetics of I_p_ were fitted exponentially for all the pH_i_ conditions tested (Fig 2F and 2G). The time constants (τ_Recvery_) were determined as 25, 30 and 14 ms at pH_i_ 7.4, 8.0 and 9.0 in the absence of Na^+^, which indicates that the peak recovery is accelerated slightly at pH_i_ 9.0 (Fig 2F and 2H). Recovery was faster in the presence of NaCl. The time constants were 13, 12.5 and 10.5 at pH_i_ 7.4, 8.0 and 9.0, respectively, indicating a slight acceleration at pH 9.0 (Fig 2G and 2I).

Next, we varied intracellular Na^+^ concentrations while the pH_i_ was fixed at 7.4. As shown in Fig 3A, the peak recovery kinetics were dependent on intracellular Na^+^ concentration. Fig 3B summarizes the τ_Recvery_ of the peak component. Recovery was slow (τ_Recvery_ = 26, 30 ms) at 0, 0.11 mM NaCl, but accelerated (τ_Recvery_ = 18 ms) at 1.1 mM NaCl and reached about 10–12 ms above 11 mM NaCl. These results indicate that late steps of the photocycle of KR2 are affected by intracellular Na^+^.

We compared off-kinetics value (after shutting of the LED), τ_off LED_, under various NaCl concentrations (Fig 3C). The τ_off LED_ were determined as about 14 ms at 0, 0.11 and 1.1 mM NaCl, while they are slightly smaller (about 10 ms) at 11 and 110 mM NaCl.

We then compared photocurrent density of the peak component (pA/pF) (Fig 3D). The current density of I_p_ ranged from about 4.7 to 5.6 pA/pS at 0 to 11 mM NaCl but showed no statistically significant differences. On the other hand, current density reached about 6.5 pA/pS at 110 mM NaCl (Fig 3D) which was significantly larger than that at 1.1. mM (p<0.05) and at 11 mM (p<0.05) of NaCl. The steady state component (I_ss_) showed no significant differences among all the conditions tested (~1 pA/pS).

### Probing for the M-intermediate in the photocycle

Previous studies demonstrated that the M-intermediate of H^+^ pumping rhodopsins can be probed by blue-light illumination on top of green or red light in electrophysiological recording [13, 14, 21, 22]. As the M intermediate exhibits a blue-shifted λ_max_, 13-*cis* retinal could be re-isomerized by blue light absorption and it directly turns into the dark state without a net proton pump. Thus, the photocurrent is quenched by blue light illumination on top of green (or yellow) light, indicating that the quenching amplitude reflects accumulation of the M-intermediate. To assess voltage dependency of the M-intermediate accumulation during green illumination in KR2, we tested the effect of a blue light pulse (54 mW/mm^2^) on top of a strong (4.6 mW/mm^2^) or weak (0.49 mW/mm^2^) green background illumination. Fig 4A–4D represent typical photocurrent traces in the absence or presence of intracellular NaCl whereas extracellular NaCl was fixed at 140 mM. On top of a green light (4.6 mW/mm^2^), blue light (54 mW/mm^2^) induced a negative transient peak current followed by a slight reduction (only 2–3 pA) of the steady state current (Fig 4A). When the blue light was shut off, a positive transient peak current appeared. The quenching effect of the steady state current by the blue light was more apparent in the presence of 110 mM NaCl inside (Fig 4B).

We then investigated the blue light effect on top of a weak green light (0.49 mW/mm^2^) (Fig 4C and 4D). In the absence of NaCl inside, no quenching of the steady state current was observed. However, a small transient peak was seen (Fig 4C inset). Notably, the direction of the peak current depends on membrane voltage. In the presence of NaCl inside, a large positive peak current was induced by blue light whereas the steady state current level was unchanged (Fig 4D). Fig 4E–4H shows the voltage dependency of blue light. Under a strong green light, we observed no obvious voltage dependency of the amplitude of the blue light effect in the absence and presence of intracellular NaCl (Fig 4E and 4F). On the other hand, voltage dependency was elucidated under weak green light. The transient peak upon blue light illumination was directed inwardly at -60 mV in the absence of NaCl and reversed at about -10 mV (Fig 4G). An outwardly directed current was observed at a more positive voltage. Such a tendency was seen in the presence of intracellular NaCl, although the current direction was not reversed in which the blue light-induced peak current was positive at all the measurable membrane potentials (-60 to +40 mV) and increased as the voltage rose (Fig 4H).

### Flash laser electrophysiology

We measured the KR2 photocurrent under a single-turnover by using a flash laser (5 ns, 532 nm) (Fig 5). A laser-evoked large photocurrent was detected from KR2-expressed cells in the absence and presence of Na^+^ intracellular solutions (Fig 5A and 5B). The photocurrent reached over 200 pA and decayed into the baseline with a tau-off value (τ_off Laser_) of 0.37 ms in the absence of Na^+^ and 0.56 ms in the presence of Na^+^. Fig 5C summarizes the off-kinetics values from the LED and the flash laser recordings. The tau-off value obtained by laser stimulation (τ_off Laser_) was more than 10-fold smaller than observed by LED stimulation (τ_off LED_) regardless of the NaCl.

### Light-induced silencing of neuronal activity

The application of KR2 for neuronal silencing was already demonstrated both *in vitro* and *in vivo* [5, 9–11]. In this study, we further assessed the silencing effect in more detail in cultured cortical neurons (Fig 6). Under the current clamp mode, the resting potential was -74.8±0.99 mV in the KR2-expressing neurons (n = 33). Current injection (200 pA) evoked action potentials (Fig 6A). Illumination at 532 nm successfully inhibited neuronal excitation, reproducing previous studies (Fig 6B). The inhibitory effect was then tested with various injected currents. We found that illumination completely abolished neuronal spiking in the 100 pA injection (Fig 6D) while no effect was observed without illumination (Fig 6C). Spike frequency was effectively reduced at the injected current up to 300 pA (Fig 6D). We finally tested light-power dependency for neuronal inhibition (Fig 6E). The dependence of the silencing efficiency on the light intensity was measured when neurons were excited by a 150-pA injection, in which spike frequency was suppressed by 64% at 0.5 mW/mm^2^ light and reached about 80% at over 1.5 mW/mm^2^ (Fig 6E).

## Discussion

Attempts were made in this study to gain a more precise understanding of the biophysical properties of sodium-pumping rhodopsin and to assess the applicability of KR2 in optogenetics research. Since the discovery of KR2, only the limited number of electrophysiological studies have been reported so far, despite a number of intensive studies by spectroscopy and X-ray crystallography. This low number was partially due to the low expression level of NaRs in cultured mammalian cells, making the detailed study of ion transporting properties difficult. Recently, we improved the expression of several NaRs and observed a relatively larger photocurrent in a whole-cell patch clamp [9]. Such efforts were also made by Hoque *et al*. and Grimm *et al*. [10, 11].

In the absence of Na^+^ in solution, NaRs transport H^+^ at a high rate. This was demonstrated by the pH electrode experiment and by UV-Vis spectroscopy [2]. In contrast, the ion transport properties in the presence of Na^+^ would be more complicated. Although Na^+^ pumping has been demonstrated by pH electrode measurements, a fraction of the pump might involve H^+^ transport. Thus, we sought to determine the ion transport property of NaR by electrophysiological measurements. Transported ion species could be determined by the substrate binding affinity as a usual enzymatic function. Therefore, we sought to compare the photocurrent amplitude and kinetics among various intracellular Na^+^ concentrations and pH_i_. To avoid effects in the extracellular Na^+^ pocket, the bath solution contained NaCl in all measurements [5, 6].

The current amplitude in the presence of 110 mM NaCl was larger than at lower Na^+^ concentrations (1.1 and 11 mM) (Fig 3D). This indicates that Na^+^ transport is increased at higher Na^+^ level. On the other hand, the current amplitude at 0 and 0.1 mM NaCl is larger than that at 1.1 and 11 mM NaCl, and reached to the same level as that at 110 mM NaCl (Fig 3D). This suggests that H^+^ pumping is enhanced at lower NaCl.

Current shape exhibited no significant differences between the H^+^ and Na^+^ pump conditions (Fig 1A and 1B). However, a difference between H^+^ and Na^+^ transport was found in the I/V plot of the steady state current. Steady state amplitude increased as voltage rose under the H^+^ pump condition (Fig 1G) which is similar to the characteristics of H^+^ pumping rhodopsins [13, 14, 16, 23]. This is interpreted as a photocycle acceleration at a higher membrane voltage, i.e., ion pumps can work faster under a smaller electrochemical load or under an energetically downhill condition.

Surprisingly, the steady state amplitude was markedly reduced at a higher voltage in the Na^+^ pumping mode (~30% reduction / 100 mV) (Fig 1H and S2J and S2K Fig) which was not consistent with the electrochemical gradient of Na^+^ to efflux the cell. We here consider photoisomerization of the M state, which transformed directly into the dark state without net Na^+^ transport (no-pumping cycle). Thus, when the no-pumping cycle is favored at a higher voltage, the photocurrent amplitude is decreased at higher membrane potential. This idea is supported by the blue light effect (Fig 4). Reduction of the photocurrent following the application of blue light on top of green light is due to photoisomerization of the M intermediate into the dark state without net ion transport. The blue light effect was more obvious under strong green light, indicating the accumulation of M intermediate. Taking into account this idea, the reduction of I_ss_ in stronger green light, as shown in Fig 1D, is explained by photoisomerization of the M state by green light itself. Voltage dependency of the equilibrium between M1 and M2 was already reported in H^+^-pumping bacteriorhodopsin [22].

**Fig 7:**
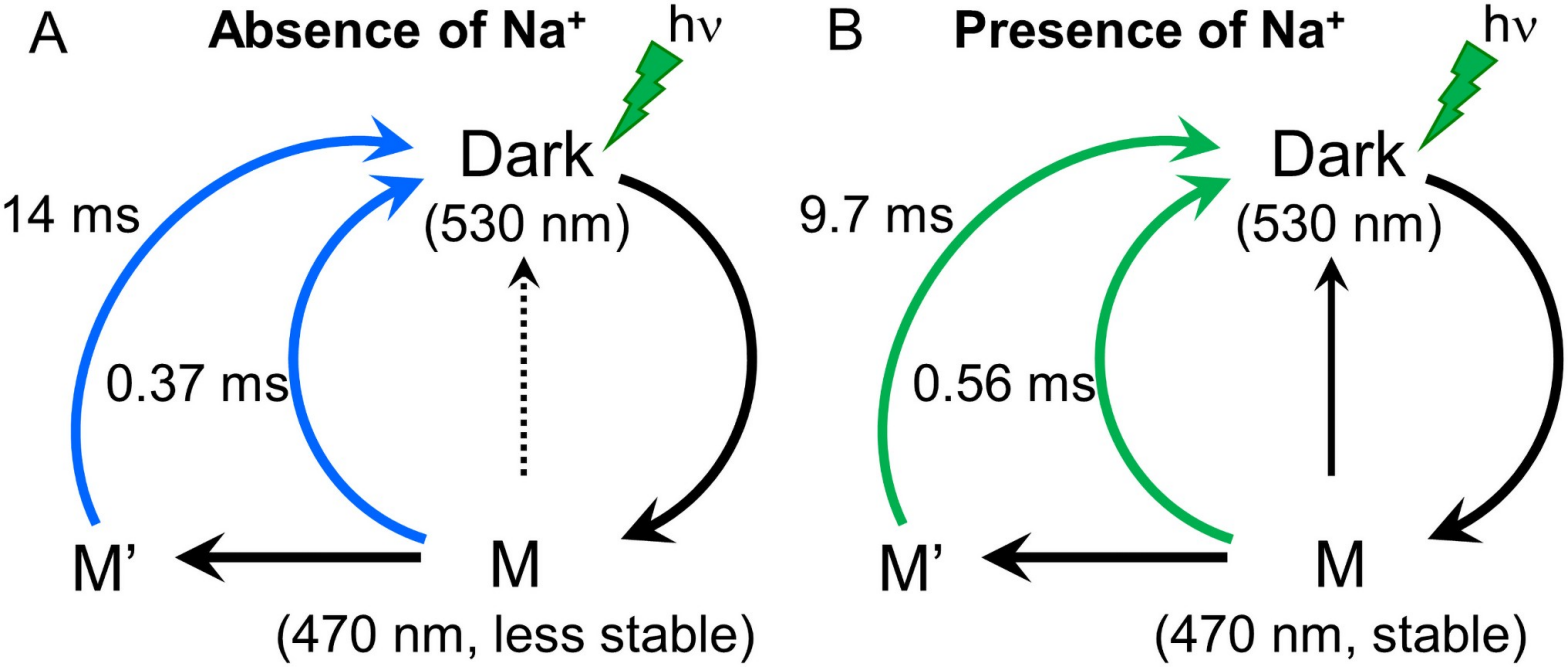
Reaction scheme of the photo/transport cycle of KR2. A, The cycle is initiated by green light absorption of the Dark state to form M-intermediate. The M-intermediate decays in to the dark state with a time constant of 14 ms (τ_off LED_). KR2 transport H^+^ indicated in blue arrows. Blue light illumination triggers a photoisomerization of M-intermediate, which result in a no-transport cycle indicated in a dotted line. B, Similar cycle can be proposed for Na^+^ transport with time constant of 9.7 ms (τ_off LED_). The no-pump cycle by blue light (black arrow) is favored in the presence of Na^+^ compared to the absence of Na^+^, because M-intermediate is more stable according to the result in Fig 4.

Single-turnover ion transport recording by using a flash laser provided additional kinetic parameters, τ_off Laser_ (Fig 5). Importantly, the τ-off value obtained by a single-photocycle (τ_off Laser_) and those investigated by continuous LED (τ_off LED_) are different by one order of magnitude. This might indicate two distinct ion release pathways in KR2. The schematic model of ion transport of KR2 was depicted in Fig 7.

Grimm *et al*. reported a flash laser-induced photocurrent from an enhanced version of KR2 (eKR2) [11]. We essentially obtained the same photocurrent properties in the presence of a high concentration of Na^+^ inside. The photocurrent decay was better fitted by a single exponential function in our case, whereas Grimm *et al*. determined three kinetic parameters.

### Optogenetics

The optical silencing effect of the cultured cortical neurons again revealed the effectiveness of KR2 for optogenetics applications in which 0.5 mW/mm^2^ light dramatically reduced spike generation (Fig 6E). The effect was already saturated at 1.5 mW/mm^2^, which agrees well with the power dependency of the I_ss_ of Na^+^ transport (Fig 1D). To gain a function for optogenetics application, it might be necessary to increase I_ss_ by accelerating the photocycle speed of KR2 by mutagenesis or by other means.

## Supporting information

S1 Fig KR2 photocurrent induced by 470 nm light in ND7/23 cells. Representative photocurrent at 0 mV in the absence (A) and presence (B) of NaCl in intracellular solution while the extracellular solution contained NaCl. 470 nm light (54 mW/mm2) was illuminated for 200 ms as the blue bar indicates. C and D, Light power dependency on the peaks (Ip) and steady state (Iss) photocurrent, in the absence and presence of NaCl intracellular solution, respectively (n = 7). E and F, Current-voltage relation (I/V plot) for the peak current (n = 12, 9). The currents were normalized to the value at 0 mV. G and H, Current-voltage relation (I/V plot) for the steady state component. The currents were normalized to the value at 0 mV (n = 12, 9).
(TIF)

S2 Fig Current-Voltage relation (I-V plot) under various ionic conditions. 532 nm LED light (25 mW/mm2) was illuminated to activate KR2. Extracellular solution contained 140 mM NaCl at pH 7.4 for all conditions. A-F, I-V plot in the absence of intracellular Na+. A-C: peak current. D-F: steady state current. A and D: pHi = 7.4; B and E: pHi = 8.0; C and F: pHi = 9.0. G-L, I-V plot in the presence of intracellular Na+. G-I: peak current. J-L: steady state current. G and J: pHi = 7.4; H and K: pHi = 8.0; I and L: pHi = 9.0. A and D: n = 7; B and E: n = 6; C and F: n = 3; G and J: n = 5; H and K: n = 5; I and L: n = 3.
(TIF)
