# Supplementary figures and images for: Ion transport activity and optogenetics capability of light-driven Na+-pump KR2
Source: PLoS One. 2021 Sep 10;16(9):e0256728. doi: 10.1371/journal.pone.0256728 (PMC8432791; doi:10.1371/journal.pone.0256728)

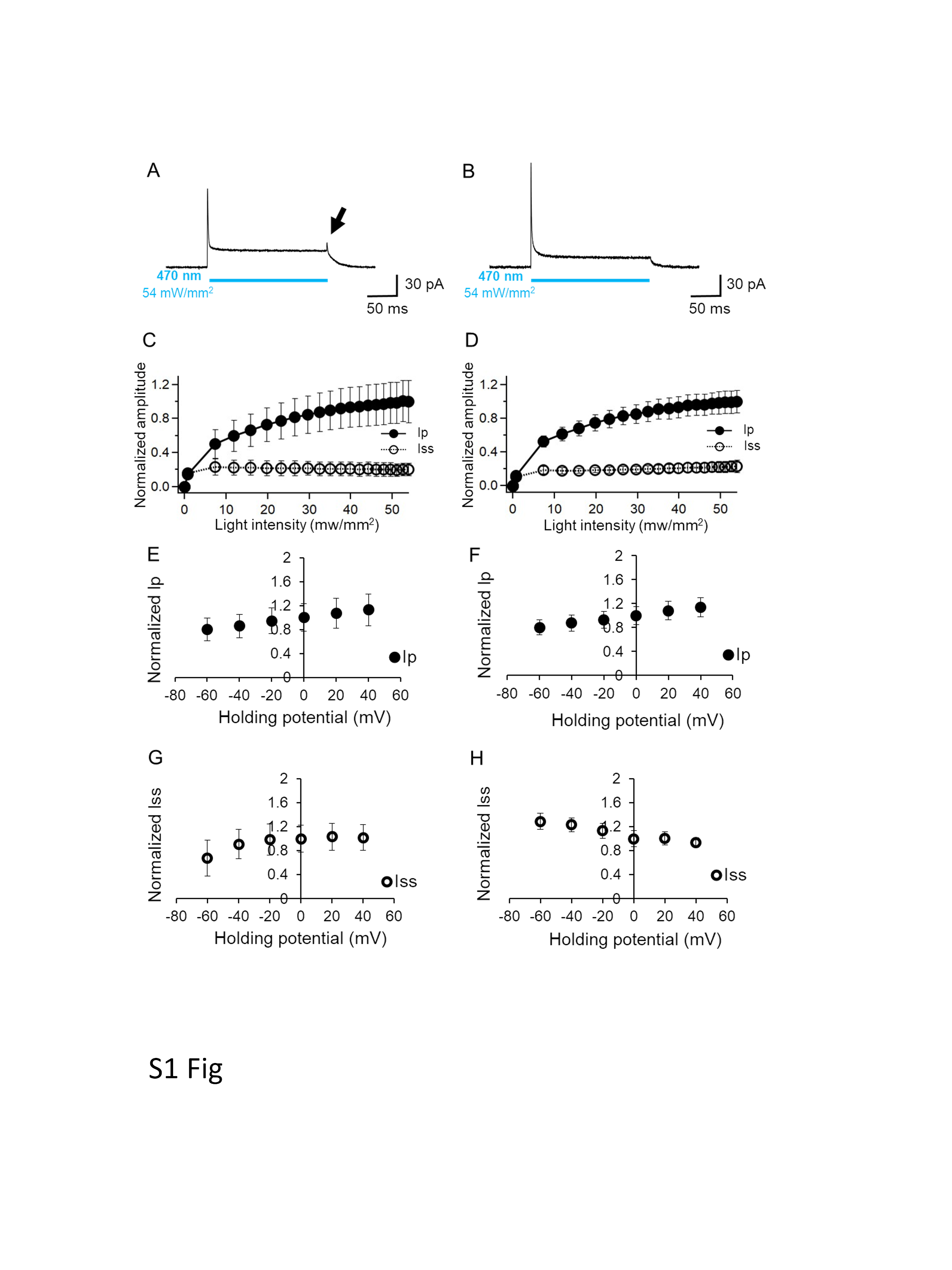

Supplement: S1 Fig — Representative photocurrent at 0 mV in the absence (A) and presence (B) of NaCl in intracellular solution while the extracellular solution contained NaCl. 470 nm light (54 mW/mm2) was illuminated for 200 ms as the blue bar indicates. C and D, Light power dependency on the peaks (Ip) and steady state (Iss) photocurrent, in the absence and presence of NaCl intracellular solution, respectively (n = 7). E and F, Current-voltage relation (I/V plot) for the peak current (n = 12, 9). The currents were normalized to the value at 0 mV. G and H, Current-voltage relation (I/V plot) for the steady state component. The currents were normalized to the value at 0 mV (n = 12, 9). (TIF) [file pone.0256728.s001.tif]

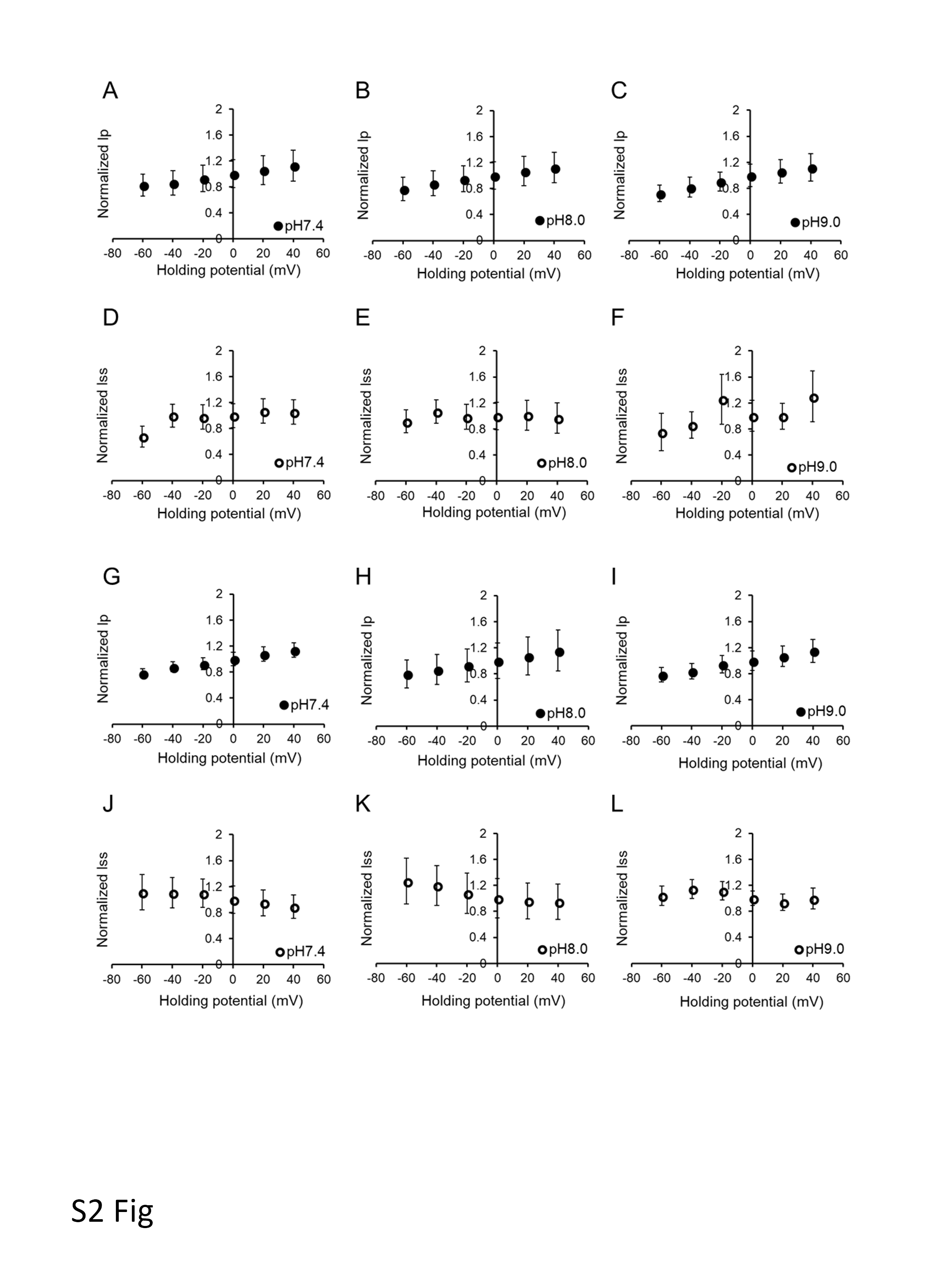

Supplement: S2 Fig — 532 nm LED light (25 mW/mm2) was illuminated to activate KR2. Extracellular solution contained 140 mM NaCl at pH 7.4 for all conditions. A-F, I-V plot in the absence of intracellular Na+. A-C: peak current. D-F: steady state current. A and D: pHi = 7.4; B and E: pHi = 8.0; C and F: pHi = 9.0. G-L, I-V plot in the presence of intracellular Na+. G-I: peak current. J-L: steady state current. G and J: pHi = 7.4; H and K: pHi = 8.0; I and L: pHi = 9.0. A and D: n = 7; B and E: n = 6; C and F: n = 3; G and J: n = 5; H and K: n = 5; I and L: n = 3. (TIF) [file pone.0256728.s002.tif]
